# Inferring causal relations from observational long-term carbon and water fluxes records

**DOI:** 10.1038/s41598-022-05377-7

**Published:** 2022-01-31

**Authors:** Emiliano Díaz, Jose E. Adsuara, Álvaro Moreno Martínez, María Piles, Gustau Camps-Valls

**Affiliations:** Image Processing Laboratory (IPL), Universitat de València, Valencia, Spain

**Keywords:** Carbon cycle, Hydrology, Statistical physics, thermodynamics and nonlinear dynamics, Statistics

## Abstract

Land, atmosphere and climate interact constantly and at different spatial and temporal scales. In this paper we rely on causal discovery methods to infer spatial patterns of causal relations between several key variables of the carbon and water cycles: gross primary productivity, latent heat energy flux for evaporation, surface air temperature, precipitation, soil moisture and radiation. We introduce a methodology based on the convergent cross-mapping (CCM) technique. Despite its good performance in general, CCM is sensitive to (even moderate) noise levels and hyper-parameter selection. We present a robust CCM (RCCM) that relies on temporal bootstrapping decision scores and the derivation of more stringent cross-map skill scores. The RCCM method is combined with the information-geometric causal inference (IGCI) method to address the problem of strong and instantaneous variable coupling, another important and long-standing issue of CCM. The proposed methodology allows to derive spatially explicit global maps of causal relations between the involved variables and retrieve the underlying complexity of the interactions. Results are generally consistent with reported patterns and process understanding, and constitute a new way to quantify and understand carbon and water fluxes interactions.

## Introduction

**Figure 1:**
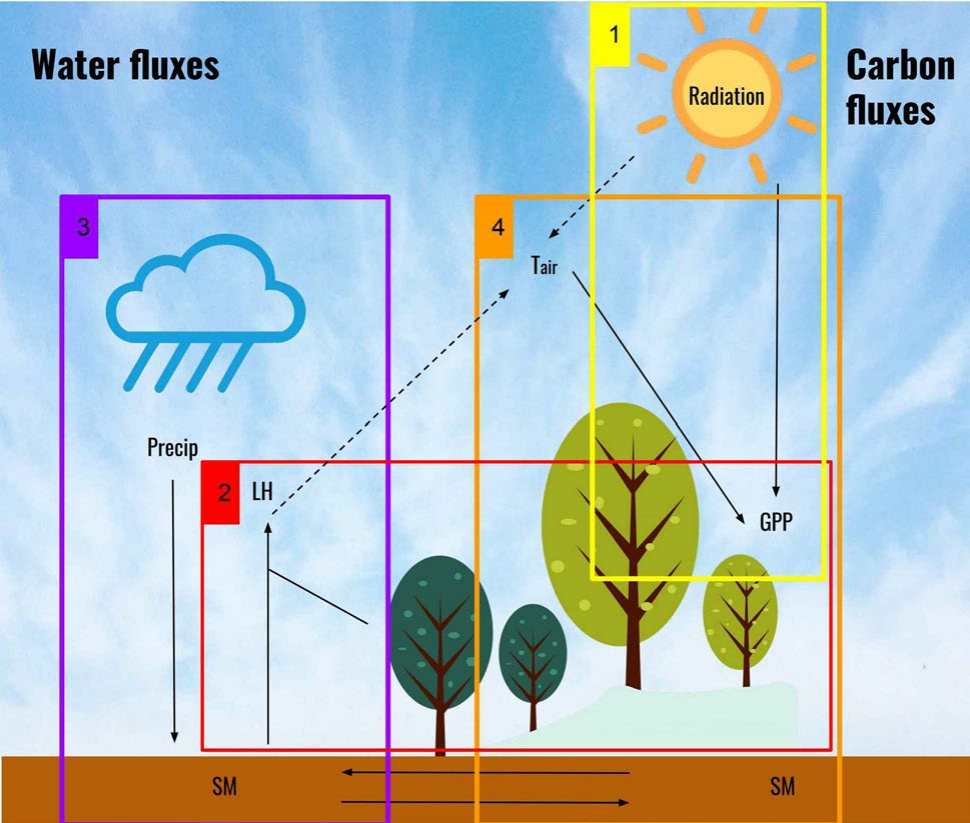
The causal dissection of the coupled carbon-water fluxes in the Earth system. Boxes indicate the variable relations studied in this paper with the proposed causal inference methodology. Dashed arrows are only implicitly considered in this work.

The Earth is a highly complex, dynamic, and networked system where very different physical, chemical and biological processes interact in and across several spheres. Land and atmosphere are tightly coupled systems, which interact at different spatial and temporal scales, cf. Fig. 1. Radiation, as the primary energy source, constitutes a clear driver of many processes and variability on Earth^1^, and directly impacts vegetation productivity, temperature and moisture, cf. Fig.1[box 1]. Precipitation patterns largely govern atmosphere and soil moisture, cf. Fig.1[box 3]. Actually, soil moisture (SM) is coupled with the atmosphere and influences climate on daily to seasonal time scales^2^. Water fluxes between the soil and the atmosphere are regulated by both land-atmosphere exchanges and large-scale atmospheric circulation patterns^3–7^, cf. Fig.1[boxes 2 and 3]. Evapotranspiration (ET) is the combined measure of evaporation and transpiration also known as the latent heat flux (LH) when it is expressed in energy as the fundamental units instead of mass. ET is tightly coupled with vegetation photosynthesis (gross primary productivity, GPP) being both ET and GPP the two dominant processes in global land, water and carbon cycles^8,9^, see Fig.1[boxes 2, 3, 4]. Additionally, components of ET are indirectly related with GPP too, mostly due to vegetation cover changes^9^. Of course, there are many other variables involved, like background wind, which may also mediate the SM-precipitation feedback, cf. Fig.1[boxes 2 and 3], and other energy, carbon, and water fluxes like net ecosystem exchange (NEE), ecosystem respiration (ER), and sensible heat (SH), which mediate in the land-atmosphere interactions and flux synchronization processes. However, the type of causal relations involved, and the time and spatial scales of interactions among these variables, are still uncertain, which limits Earth system modeling and understanding and prevents an optimal management of water and carbon resources.

The main challenge to quantify such relations globally comes from the lack of sufficient in-situ measurements, and from the fact that some of these variables are latent and not directly observable with remote sensing systems. One can, for example, measure SM but not GPP directly. As an alternative, many studies have relied on model simulations to investigate SM-precipitation^10^, GPP-SM^11^ and ET-SM relations^12,13^, to name just a few. Use of simulation models to understand real world processes, however, involves important challenges such as misspecification, oversimplification and variability across models, as well as the major shortcoming that the sign and strength of the simulated feedback relations often vary across models. To address the data scarcity issue, satellite-derived remote sensing products offer an alternative pathway. Earth observation capabilities have been greatly enhanced in the last decades and a plethora of satellites are now available, which offer great opportunities to estimate key parameters of the land, ocean and atmosphere. Nevertheless, canonical approaches to study variable relations often rely on regression and statistical association (correlation) techniques, which obviate the causal relations among variables. Furthermore results are often compromised by nonstationarities, strong autocorrelation functions and spurious correlations among variables. Causal relations among the different processes still remain largely unknown.

In this context, *causal inference* provides the proper mathematical framework to discover and explain the causal structure of the system^14–16^. Within this framework a variable *X* is the cause of another variable *Y* if intervening on *X*, necessarily affects the variable *Y* but, conversely, intervening on *Y* leaves *X* intact. Very often, interventions in the system are not possible because of ethical, practical or economical reasons. Then *observational* causal inference comes into play to extract cause-effect relationships from multivariate datasets, going beyond the commonly adopted correlation approach, which merely captures associations between variables. Causal discovery is nowadays an active field of research in remote sensing^17^, Earth^16^ and Climate^18^ sciences. Several methods are available to infer causal graphs from observational data. Granger causality (GC)^19^ is the most widely used approach in Earth and climate sciences to quantitatively identify causal relations between time series. Nevertheless, GC approaches perform poorly when applied to systems involving non-stationary or nonlinear processes and deterministic relations, especially in dynamic systems with weak to moderate coupling. To resolve the above issues, we rely on the convergent cross-mapping (CCM) method^20^, which is a nonlinear state-space method to recover the causal dynamics without the strong assumptions of linearity and stationarity. CCM evaluates the reconstruction of variable’s state spaces (${\mathcal M}_x$ and ${\mathcal M}_y$) using time embeddings, and concludes that $X\rightarrow Y$ if points on ${\mathcal M}_x$ can be predicted using nearest neighbors in ${\mathcal M}_y$ more accurately as more points are used (see Methods section). The CCM method was extended to deal with causal relations operating at different time-lags (though not instantaneously)^21^, and was applied to derive causal relations between temperature and greenhouse gases^22^, the sensitivity of the carbon cycle to tropical temperature variations^23^, and to scrutinize the relations between SM and precipitation^8^. However, even the extended CCM is sensitive to moderate noise level and hyperparameter selection, as previously shown in^24^. CCM also suffers from false detections in cases of strong, unidirectional variable coupling as reported elsewhere^21^. To address the issues of hyperparameter selection and noise sensitivity, we present robust CCM (RCCM) which relies on bootstrap resampling through time and the derivation of more stringent cross-map skill scores. Secondly, we combine the RCCM method with the information-geometric causal inference (IGCI)^25^ method to address the problem of strong and instantaneous variable coupling (see Methods section). The proposed method not only allows to infer both weak and strong causal relationships between the variables, but also provides a systematic approach to estimate the embedding dimension and thus derive spatially explicit global maps of causal relations between variables. We illustrate its performance using long-term carbon and water flux records to study the four subsystems in Fig. 1, involving main land and water fluxes relations. In particular, we use six different biosphere and atmosphere global gridded products, which are collected and curated in the Earth System Data Lab (ESDL). More details about the primary sources of information are given in the Materials section. After the homogenization, the dataset shares a common spatio-temporal grid of $0.25^\circ$ in space and 8 days in time, spanning 11 years from 2001 to 2011. We study several relevant causal problems involving photosynthesis and radiation, strong bidirectional coupling of carbon and latent heat fluxes, and the problems involving precipitation and moisture.

## Results

We show the ability of the proposed methodology to infer causal relations from observational time series in four case studies characterizing key land and atmosphere interactions, cf. Fig.1.

### The case of photosynthesis and radiation

Let us start with a clear example of strong instantaneous coupling where (even the extended) CCM^21,22^ fails: the unidirectional case of photosynthesis (i.e. GPP) driven by radiation, cf. Fig.1 [box 1].

**Figure 2:**
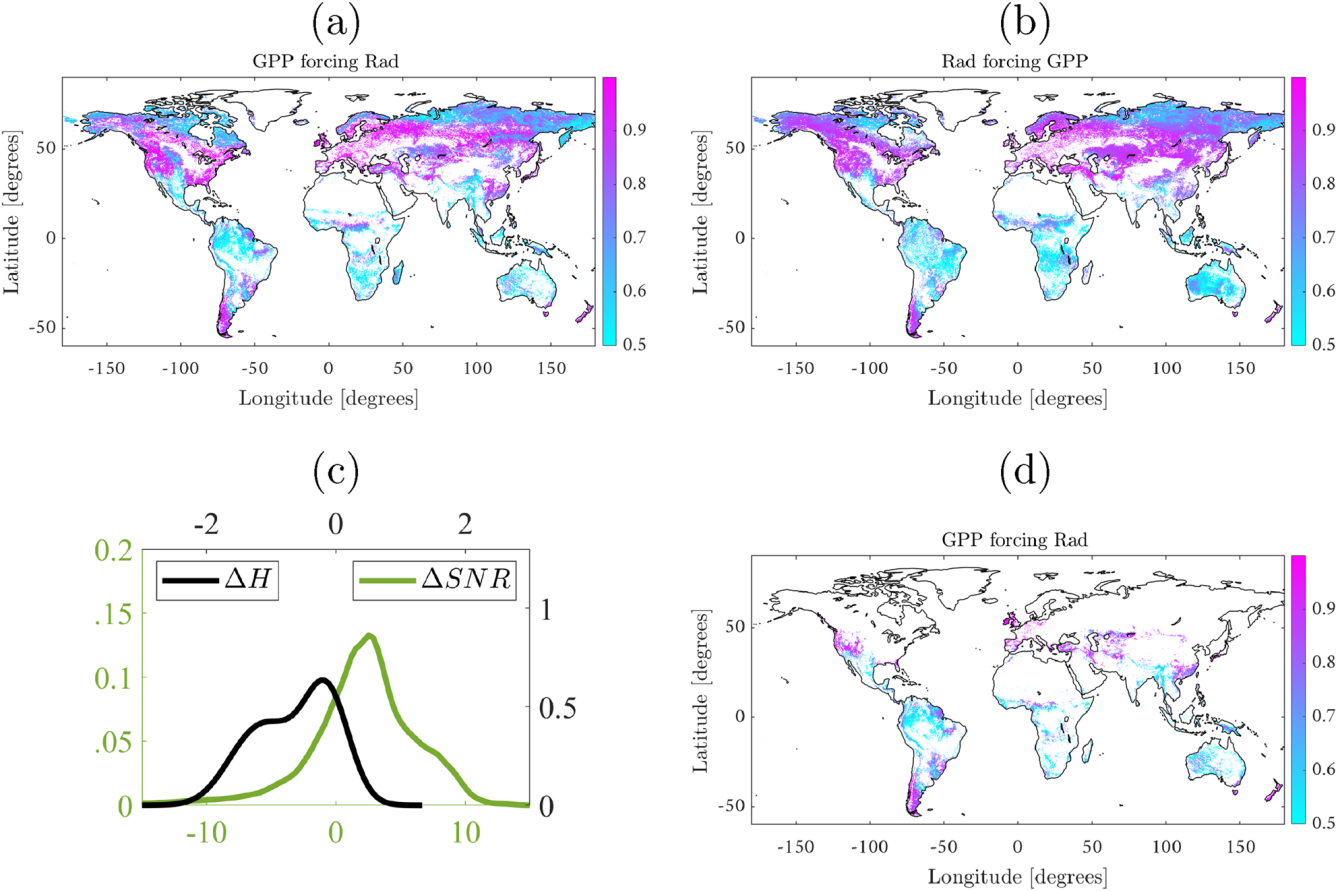
Top: (**a**) A significant and unrealistic widespread forcing of GPP over radiation is found by the CCM due to the strong immediate forcing of (**b**) radiation over GPP. Bottom: Signal-to-noise ($\Delta$SNR) [dB] and entropy ($\Delta$H) differences between GPP and radiation (**c**). The proposed methodology corrects CCM by taking into account entropy differences, and hence the estimated areas of forcing of GPP over radiation are reduced significantly (**d**). The figure was generated using Matlab version R2018a (https://es.mathworks.com).

The extended CCM relies on the assumption of the existence of a certain “asynchrony”, which reflects the time lag between cause and effect. This is not the case of the example presented here where, at the considered temporal resolution, radiation has an immediate and strong unidirectional forcing over GPP. Figure 2 illustrates the benefits of RCCM over the extended CCM. Figure 2a,b show the forcing strength of GPP over radiation and radiation over GPP, respectively, using the extended CCM. Figure 2d shows a more reasonable estimate of forcing strength of GPP over radiation using RCCM, while Fig. 2c illustrates the asymmetry in entropy between these two variables which helps RCCM to identify spurious forcings. Direct application of CCM leads to, not only inferring RAD$\rightarrow$GPP cf. Fig. 2a as is expected, but to unreasonably inferring GPP$\rightarrow$Rad worldwide, cf. Fig. 2b. Our proposal combines a robust version of CCM and IGCI, see Methods section, which is especially well-suited to moderate noise levels and instantaneous causal interactions. IGCI exploits asymmetry in the entropy between the two variables, shown in Fig. 2c, to identify the correct causal relation. The incorrect GPP$\rightarrow$Rad inference is corrected with the application of RCCM in combination with IGCI, which essentially masks strong (and simultaneous) couplings between the variables, removing around 84% of the false detections, see Fig. 2d.

Radiation is found to be an effect only in very sparse regions, threshold cross-map skill $\rho >0.8$. Even with the reduced number of anti-causal detections, our proposal identifies GPP causing radiation in tropical and cloudy regions (Amazonia), rainy regions (southern China), as well as very dry ones (Mexico, Australia and the Sahel). This could be because an increase in GPP as a result of more water availability and vegetation growth enhances LH, which moistens the atmosphere and could affect precipitation regimes, cloud cover, and thus, radiation. Additionally, in very wet regions, such as the Amazon, soil moisture rarely affects stomata closure and, under these circumstances, an increase in GPP affects both, latent and sensible fluxes. This increase in sensible heat leads to a deeper boundary layer and reduced cloud cover, which in turn affects incoming PAR^26^. Concomitantly, patterns of association emerge in regions with high variance/entropy and high cloud coverage, like the tropics and Amazonia in particular, associated to large GPP and radiation too. The identified patterns are somewhat related to the cloud feedbacks to climate (e.g., cooling and rainfall) which typically carry over large uncertainties. Regionally, cloud scaling factors are typically very low for boreal and tropical forests, where cloud cover is too dense and limits plant photosynthesis^27^.

### Assessing the strong bidirectional coupling of carbon and latent heat fluxes

Let us now study the detection of causal links when the coupling is bidirectional and strong, a well-known problem with the standard CCM. This is the case of LH and ET, which are associated with the exchange of energy and water, respectively, a key process describing soil water depletion worldwide, cf. Fig.1[box 2]. This process connects land surfaces and the atmosphere, and interacts with land carbon fluxes (e.g. GPP) and the nitrogen cycle. Water and carbon fluxes in plants are linked by stomata control during the photosynthesis process, which optimizes carbon gain while minimizing transpiration water loss^28,29^.

**Figure 3:**
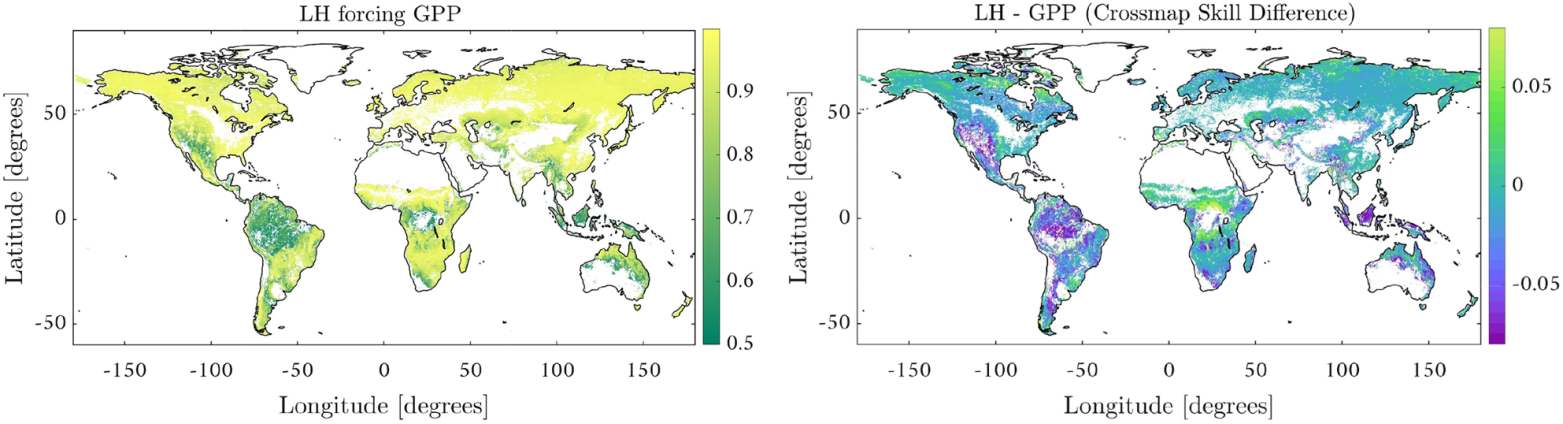
Results over two strongly coupled pair of variables (LH and GPP). Not only are the cross-map skills ($\rho$) really high in general (left figure), but also the link between these variables is further confirmed by the fact that both cross maps skills are almost identical as can seen by the low differences between them (right figure). The figure was generated using Matlab version R2018a (https://es.mathworks.com).

Figure 3 shows the strength of forcing of LH over GPP (left) and the difference between this forcing and that of GPP over LH (right). For the latter figure, note that positive differences indicate a larger forcing of LH over GPP than the other way around. We note that the RCCM has captured this strong physiological link between GPP and LH, resulting in a strong bidirectional coupling (note the almost identical high cross map skills for both variables), cf. Fig. 3. Furthermore, GPP and LH causal relations vary with climatic conditions so that in dry and wet ecosystems they appear to be less coupled. This decoupling could be explained by the significant effect in stomatal limitation to photosynthesis over high temperature regions^30^, but also by the differing response of GPP and ET to atmospheric vapor pressure deficit (VPD) changes in specific environments, such as the tropics. While ET in tropical and temperate climates is likely to show positive responses to increasing VPD (increased atmospheric demand)^31^, GPP could be negatively affected by stomata closure. In general, we observe low cross-map skill differences in Fig. 3[right], yet results suggest stronger forcing of GPP$\rightarrow$LH in high water availability regions (e.g. Amazonia) while LH$\rightarrow$GPP in cold ecosystems and transition areas (e.g. African Sahel).

### The causal relations between latent heat, precipitation and soil moisture

Let us now assess the more complex causal relations in the water cycle between LH, precipitation (Precip) and soil moisture (SM), cf. Fig.1[box 3]. We are aware of the many challenges in the detection and quantification of the soil moisture-precipitation feedback, which has been studied with CCM before^8^. Here we do not deal with this specific challenging problem, and focus instead on illustrating the performance of the proposed methodology in identifying well-known direct causal links under the already difficult strong coupling conditions. More precisely, we want to identify the relative dominance of drivers of soil moisture such as Precip (which recharges soil water storages) and LH (which represents soil water loses due to evaporation and transpiration processes).

**Figure 4:**
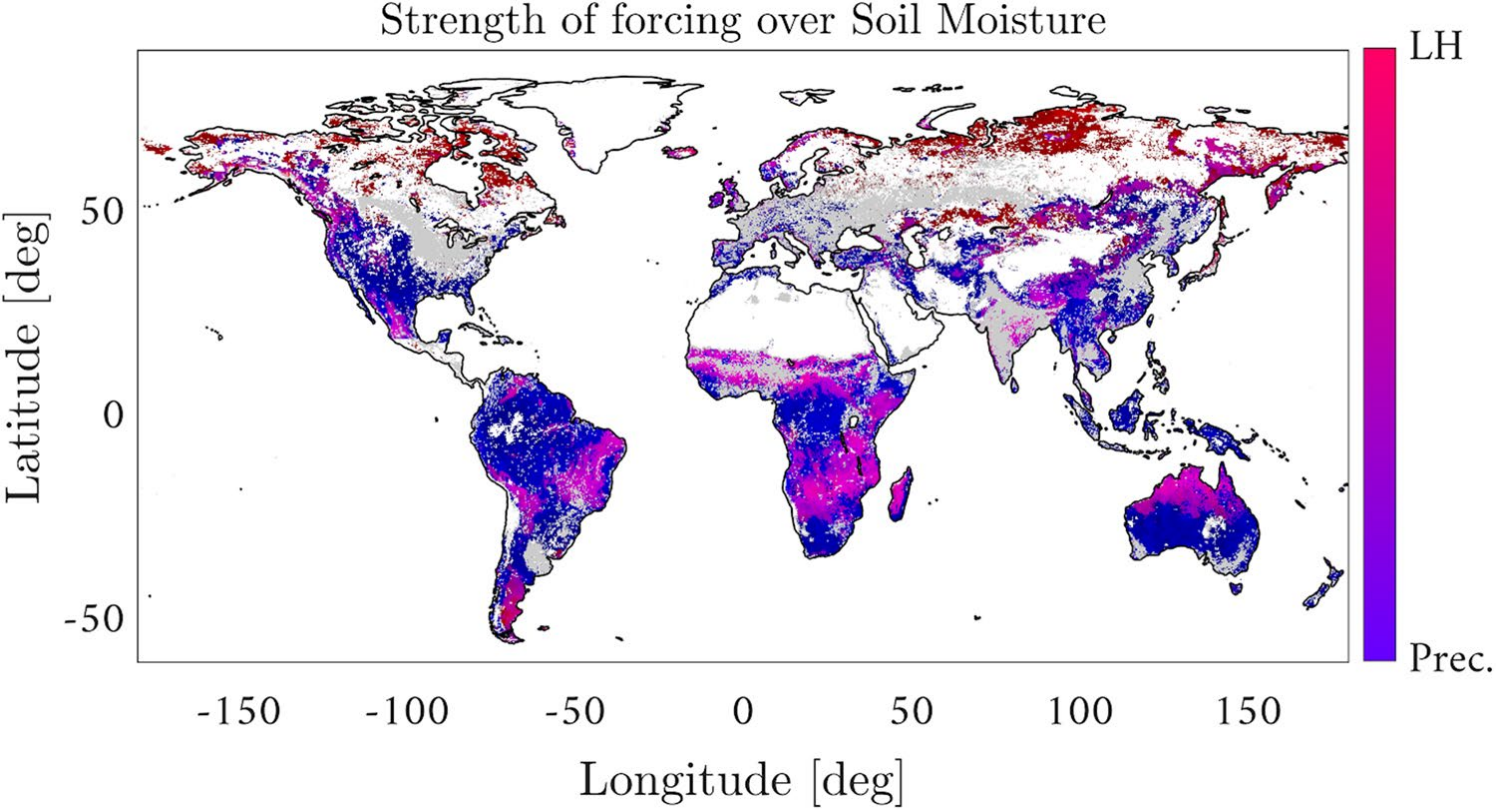
Relative forcings of precipitation and latent heat energy for evaporation on soil moisture. Reddish colors indicate a dominant effect of evaporation processes while bluish colors indicate rainfall water income as the dominant driver for soil moisture variations. The grey areas correspond to crop land areas that have been deliberately masked to avoid confounding effects related with human intervention and its effects on soil moisture. The figure was generated using Matlab version R2018a (https://es.mathworks.com).

Figure 4 maps the predominant driver of SM between LH and precipitation. Bluish regions indicate the predominance of precipitation (cross map skill for precipitation above 0.9 and below 0.4 for LH) while pinkish regions indicate the predominance of LH (cross map skill for precipitation below 0.4 and above 0.9 for LH). Figure 4 shows that the largest causal imprint of LH and precipitation in SM occurs in the tropical and subtropical regions, where the high surface temperature conducts much heat into the air above, and is lowest near the poles where the surface temperatures are much lower. The method identifies the dominant forcing of precipitation (bluish) over SM mainly in wet tropical forests and in arid and semi-arid regions such as south-western United States, south Africa, and central Australia. In dry tropical forests both LH and precipitation jointly force SM to some degree (pinkish).

Over boreal/cold ecosystems, LH emerges as the main driver of SM variability (reddish colors). This can be explained by the processes of soil thawing and freezing, which are accompanied by frequent phase transitions between soil ice and soil water, resulting in the absorption and release of latent heat^32^. In^33^, positive and negative effects of precipitation over GPP are found for different subregions across northern Eurasia. In this study, we do not evaluate precipitation directly as a potential cause of GPP since we consider soil moisture instead, in part because it is a smoother time-averaged proxy for water availability. However, over northern Eurasia, we find that soil moisture is either driven more by latent heat than by precipitation or not forced by either one. This is possibly because soil moisture is driven by melting snow more than by precipitation in these regions.

### The photosynthesis, temperature and soil moisture causal relations

**Figure 5:**
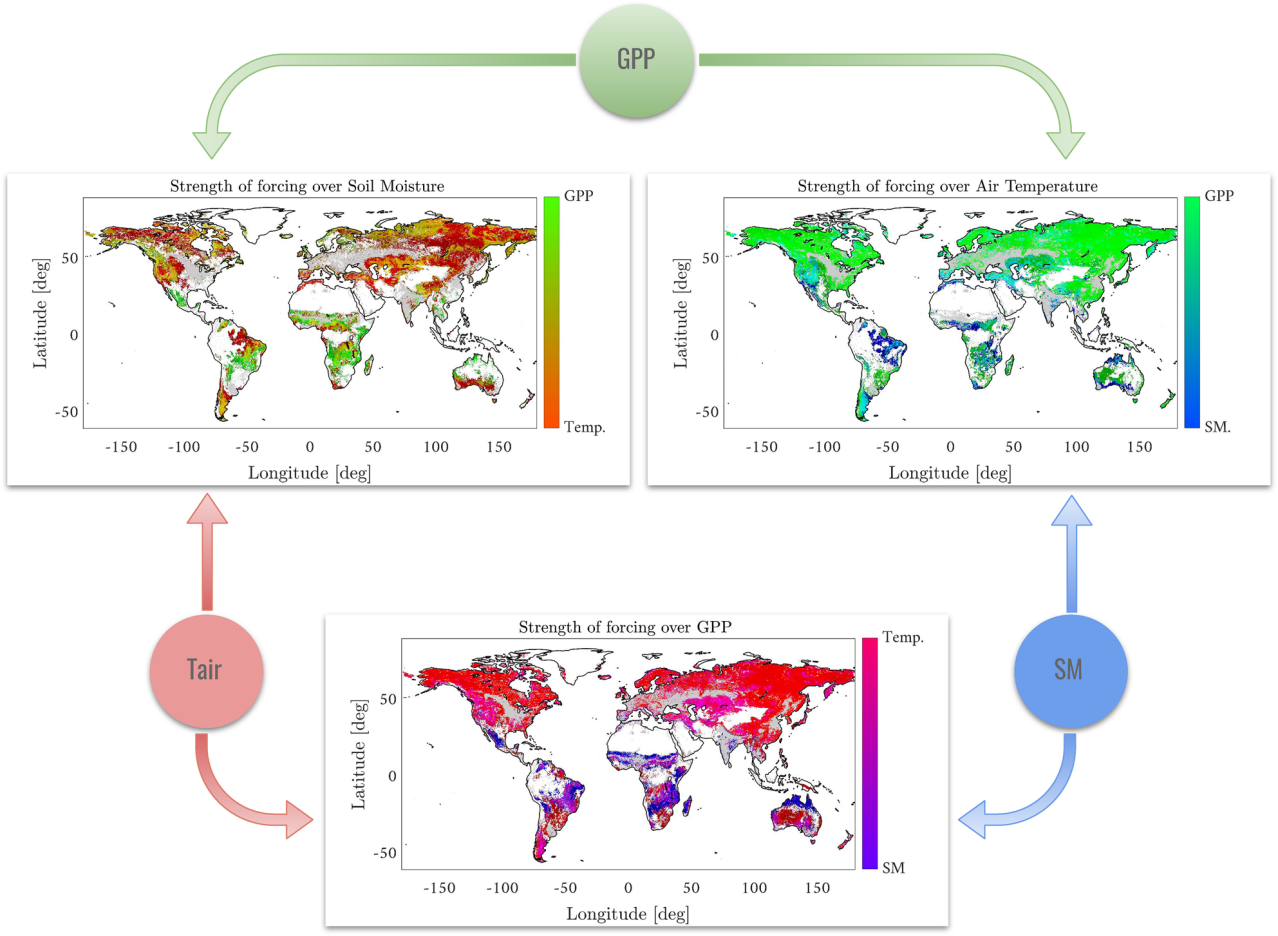
Application of the RCCM to derive causal relations between variables accounting for photosynthesis (GPP), temperature (Tair) and soil moisture (SM). Reasonable spatial causal patterns are observed for SM and Tair on GPP; GPP drives Tair mostly in cold ecosystems (probably due to changes in land surface albedo such as snow/ice to vegetation changes); SM is mostly controlled by Tair, which partially drives evaporation in water-limited regions; and GPP dominates SM. The grey areas in the images correspond with crop land areas that have been deliberately masked to avoid confounding effects due to human intervention specially on soil moisture. The figure was generated using Matlab version R2018a (https://es.mathworks.com) and Powerpoint version 2016 (https://microsoft.com).

Our final case study deals with the complex interactions of three key variables in the carbon cycle: moisture, photosynthesis and air temperature (Tair), cf. Fig.1[box 4]. For this study, we initially considered the use of both the surface and the root-zone soil moisture. While surface soil moisture is appropriate for the study of causal relations with air temperature and precipitation, root-zone soil moisture should ideally allow for a more accurate description of the forcing of SM on GPP. There is evidence, however, that at large scales, root-zone soil moisture anomalies are caused by the downward propagation of atmospheric anomalies through the surface layer and into the root-zone^34–37^. Therefore, adequate monitoring of surface soil moisture should provide the information necessary to re-construct root-zone anomalies. High correlation between the surface moisture and root moisture products is consistent with this evidence. In Materials section we provide more discussion aswell as correlation maps between surface and root moisture which support these claims. Figure 5 maps the predominant drivers of GPP, Tair and SM. For each of the three variables the figure shows which, out of the remaining two, is the dominant driver. Analogous criteria for dominance to that described for Fig. 4 is used. Reasonable causal patterns of interaction are observed in Fig. 5. Note that GPP drives Tair mostly in cold ecosystems probably due to changes in land surface albedo such as snow/ice to vegetation changes, cf. Fig.  5[bottom]. Results show important forcings of GPP on local temperature in many areas. Some attribution studies such as^33^ have also found that temperature is an important driver of GPP but did not study the reverse relationship. However, these results agree with recent large scale analyses that highlighted the impact of temporal changes on Leaf Area Index (LAI) and GPP on the surface energy budget. These complex relationships are mostly driven by radiative factors in cold climates (reduction of surface albedo and surface warming) and by turbulent energy fluxes in warmer and drier ecosystems (enhancement of latent exchange and subsequent cooling effect)^38,39^.

Soil moisture and near-surface climate are closely related to changes in air temperature. SM limits the available energy for evaporation (latent heating) and induces an increase of near-surface air temperature under dry conditions. Increased air temperature could yield higher VPD, enhanced atmospheric water demand and evapotranspiration as well as decreased soil moisture. This could plausibly explain the significant strength of forcing of air temperatures in the high latitudes. SM is mostly controlled by Tair which partially drives evaporation while GPP mainly dominates SM in water-limited regions (cf. strong LH-GPP coupling as a confounder), cf. Fig. 5 [top-left]. We note that GPP temporal variability seems to be mostly driven by air temperature, especially over northern high latitude ecosystems where cold temperatures constrain plant photosynthesis and hence plant growth.

GPP and ET are tightly related since carbon assimilation in plants is linked with water losses through transpiration^40^. Low water availability in vegetation has an important effect reducing both GPP and ET, creating a less efficient sensible heat cooling mechanism, which increases air and surface temperatures but also dries the atmosphere. Results also confirm the known observation that stronger forcing of SM over GPP is mostly located over transitional regions from wet and dry climates^4^. Interestingly, no strong forcings were found in tropical rainforest areas, indicating that in these regions GPP is mostly driven by the amount of available solar radiation and negatively impacted by high VPD values^41^.

**Figure 6:**
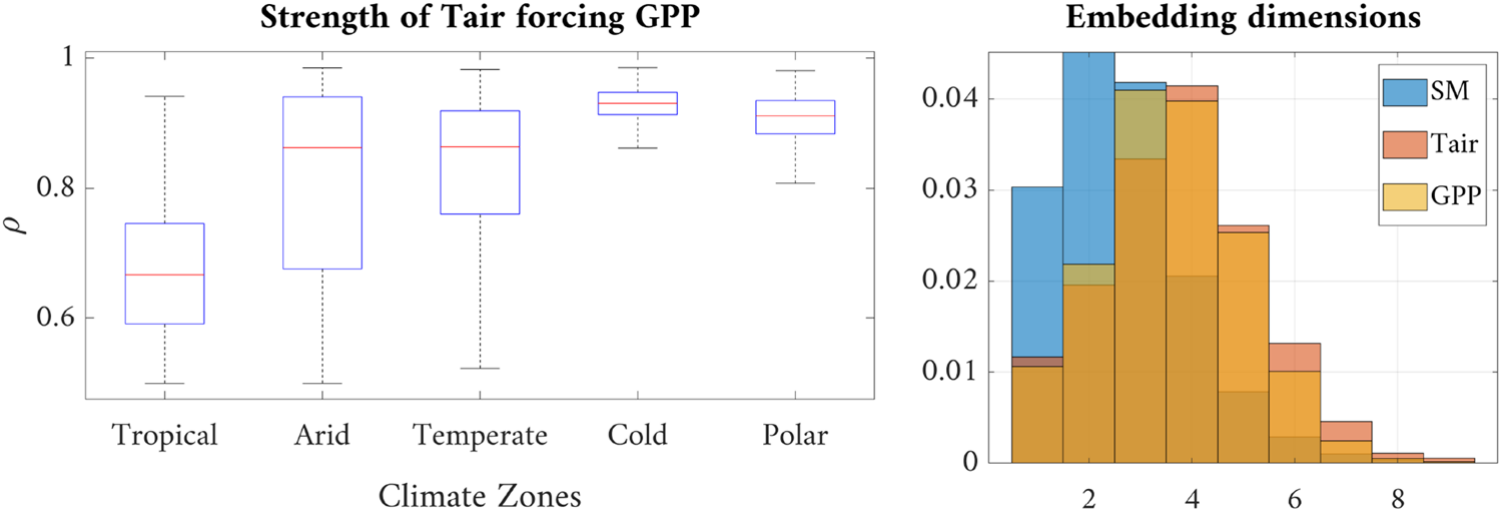
Stratified analysis of the forcing of Tair on GPP over major climate zones (left). Probability distribution of the pixel-wise dimensionality of the embedding computed with the proposed RCCM approach (right).

The proposed methodology allows us to study the strength of the coupling between variables (e.g. Tair forcing GPP) per climatic zone, see Fig. 6[top-left]. As expected, the cross-map skill increases in extreme cold and polar regions, and shows a higher spread and variability in arid, temperate and tropical regions. The methodology also allows us to characterize data complexity by looking at the robust estimation of the optimal embedding dimension, see Fig. 6[top-right]. Photosynthesis is a very plastic and adaptable process which maximizes carbon acquisition in a narrow configuration of meteorological and resource availability conditions. As a result, plants have developed protection, regulation, and acclimation mechanisms, which ultimately affect GPP in response to non-optimal scenarios^42^. This results in a more complex (high embedding dimension) temporal behaviour for GPP, specifically due to the combined impacts of physiological and meteorological drivers such as SM and Tair considered here.

## Conclusions

This study introduces a methodology based on CCM to infer causal relations from observational long-term carbon and water fluxes records. The proposed RCCM methodology can cope with strong and instantaneous coupling and moderate noise levels more efficiently. It allows causal links to be uncovered globally from a set of relevant variables in the coupled carbon-water cycles: GPP, soil moisture, precipitation, latent energy and air temperature. The approach allows one to 1) disentangle GPP-LH strong coupling by looking at bootstrapped differences, 2) capture the most relevant drivers of SM spatially, 3) detect the causal links between LH and GPP, and 4) infer forcings of SM and Tair on GPP. The method estimates the time embedding systematically, and thus facilitates the generation of spatially explicit causal impact maps. This in turn allows for the study of relations locally and to characterize the complexity of land-atmosphere interactions explicitly by a more robust estimation of the cross-map skill. Despite obtaining promising results, some further work is still needed in the future. In particular, a theoretical analysis about the combination of CCM and IGCI needs to be performed to fully characterize robustness and identifiability power. We should also note that to allow for a spatial application of our methodology, we had to rely on global gridded products, i.e. estimations of the variables of interest based on physical and/or statistical models. Any causal assumptions implicit in these models could ostensibly bias the inference of causal hypotheses. Note that, since climate change can also modify land-atmosphere coupling, more work is needed to deeply understand possible regional changes in climate feedbacks. Advances on more robust identification of causal relations will allow us to gain insights on physical processes and leave mere (and potentially spurious) correlation patterns behind.

## Materials

### Data collection

We used six different biosphere and atmosphere global gridded products, which are collected and curated in the Earth System Data Lab (ESDL). This platform includes a wide range of variables encoding atmospheric, climate, and terrestrial conditions. The uptake of atmospheric carbon dioxide by vegetation through photosynthesis is commonly referred to as Gross Primary Production (GPP) and is the largest carbon flux in the global carbon cycle. We used the FLUXCOM GPP (remote sensing dataset) which is the result of an upscaling of flux tower measurements based on multiple machine learning algorithms and satellite data as input, including the Enhanced Vegetation Index (EVI), LAI, band 7-Middle Infrared Reflectance (MIR), Normalized Difference Vegetation Index (NDVI), and Normalized Difference Water Index (NDWI)^43–45^. GPP is measured in gC m$^{-2}$day$^{-1}$, and the product spans from 2001 to 2012, with a spatial resolution of 5 arc-minutes and temporal resolution of 8 days. Another related key variable in land-atmosphere interactions is the latent heat flux (LH, measured in W m$^{-2}$), and is considered a major driver of the global hydrological cycle. LH is the flux of energy from the Earth’s surface to the atmosphere that is associated with evaporation or transpiration of water at the surface and subsequent condensation of water vapor in the troposphere. We used the harmonized LH in the ESDL^43–45^, covering the same period and spatial resolution as GPP. A crucial driver in the hidrological cycle and the vegetation productivity is the surface temperature. We used the two-metre temperature (Tair [K]) product^46^ from the ERA-Interim reanalysis product (a combination between assimilation and forecasting). The spatial sampling is approximately 80 km and temporal sampling is 6/3 hours (analyses/forecasts). The precipitation product used in this work (Precip) spans between 1980 and 2015, and was based on the Global Precipitation Climatology Project (GPCP)^47,48^. The surface soil moisture (SM) spans between 2001 and 2011 and was created by using the Global Land Evaporation Amsterdam Model (GLEAM)^49,50^, input forcing data sets from reanalyses, optical and microwave satellites and other merged sources. The data has a spatial sampling of 0.25$^\circ$ and a daily time resolution. The estimate of soil moisture corresponds to the top 10cm layer of soil. We also used the incoming surface shortwave radiation data (Rad) of the Japan Aerospace eXploration Agency (JAXA) Satellite Monitoring for Environmental Studies (JASMES) product for 2001-2015 period (available at ftp://suzaku.eorc.jaxa.jp/pub/GLI/glical/Global_05km/repro_v6/). The products are derived from Terra MODIS data with a simple radiative transfer model^51^. Spatial and temporal averaging was conducted by converting the original 5 km grid to $0.0833^{\circ }$ grids and daily to 8-day temporal resolution. Missing data in the original 5km data were replaced by mean daily values of available years.

After the homogenization, the dataset share a common spatio-temporal grid of $0.25^\circ$ in space and 8 days in time, spanning 11 years from 2001 to 2011. Stationarity is implicitly assumed as the method relies on time lag reconstruction of the time series.

### Surface vs. root moisture

Using surface moisture as a proxy of root moisture is supported by previous research in literature, for instance:

1. In^34^ it is shown that the exponential filtering of surface (0-5cm) soil moisture produces a root-zone soil moisture proxy that has as much information relevant to drought impacts on vegetation (measured via NDVI) as that contained in actual root-zone (0-40cm) soil moisture observations.
2. Analogously^35^ studies the information content of surface moisture relevant to latent heat flux. Again, surface soil moisture (or the simplistic filtering of surface soil moisture) contained as much information for surface energy flux prediction as actual root-zone soil moisture observations.
3. In^36^ it is shown that annual variations in total terrestrial water storage can be captured by smoothing and lagging surface soil moisture observations.
4. In^37^ it is shown that SMAP L2 surface soil moisture observations alone can be used to partition rainfall in runoff and ET and storage components. So, simliarly to^36^ they argue that - despite their limited vertical depth - surface soil moisture retrievals contain significant water balance information.

**Figure 7:**
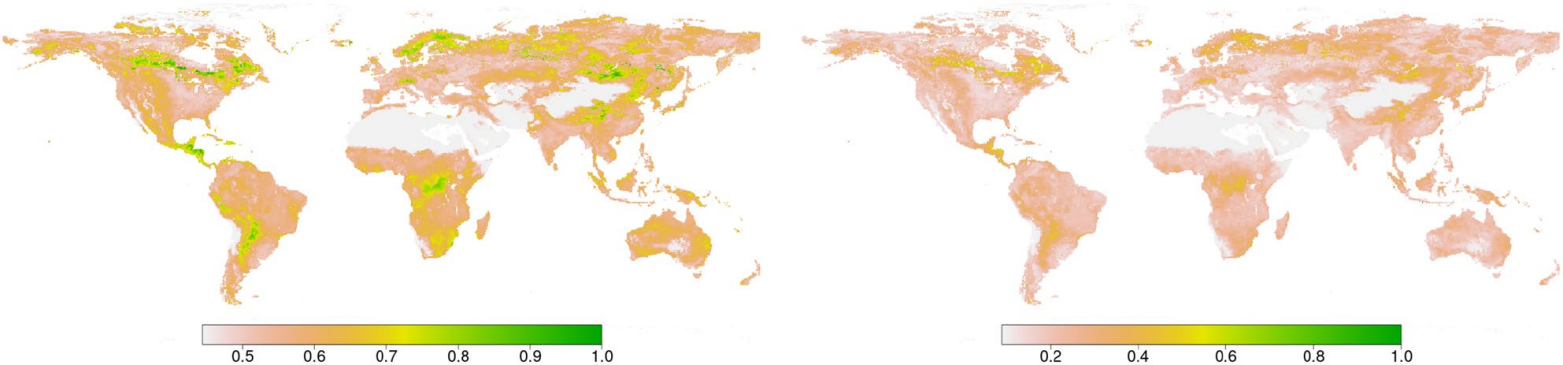
Pearson’s and Spearman’s correlation by pixel between root and surface moisture for the 11 year period used. The figure was generated using R version 3.6.1 (https://www.r-project.org/) using the raster package version 3.0-7 (https://cran.r-project.org/web/packages/raster/raster.pdf).

High correlation between the surface moisture and root moisture products as shown in Figure 7 is consistent with this evidence.

In 96.3% of pixels the Spearman’s correlation is above 0.7. Note that Spearman’s correlation is especially relevant here since CCM is a non-linear method meaning it is not sensitive to non-linear transformations of the variables.

## Methods

Many methods for causal discovery exist in the literature^14,15^ and are applied in the Earth sciences^16^. In this work, we combine two methodologies: CCM^20^ method and the IGCI approach^25^. We introduce the RCCM approach that leads to improved robustness and automatic parameter selection, and combine it with the IGCI criterion that masks out causal inconsistencies under strong couplings.

### Robust convergent cross-mapping

#### Standard and extended CCM

Convergent Cross-Mapping (CCM)^20^ is a well suited causal discovery method for systems not covered by GC: nonlinear deterministic dynamic systems with weak or moderate coupling. Relying on Taken’s theorem^52^, if *X* is causally influencing variable *Y* ($X \rightarrow Y$) then the method looks for the signature of *X* inside *Y*’s time series, i.e., information of *X* which is redundantly present in *Y*, something guaranteed by the previous theorem for causally related variables. So we can reconstruct *X* using only the information in *Y*. Therefore, two manifolds can be reconstructed from lagged coordinates of the time-series variables. From *Y*, the so-called ${\mathcal M}_Y$, used to cross map variable *X*, and denoted as $\hat{X}(t) | {\mathcal M}_Y$. The same can be done for variable *X* yielding ${\mathcal M}_X$ and $\hat{Y}(t) | {\mathcal M}_X$. If bidirectional causality exists, each variable can be estimated from the other, and cross mapping will show convergence as the number of points used for the estimation grows. Otherwise, for non-coupled variables, cross mapping will show no evidence of convergence in one of the directions and unidirectional causality can be inferred. To mitigate the problem of generalized synchrony in systems with a strong unidirectional forcing, where CCM fails, time-delayed causal interactions was introduced in^21^. For a given causal direction, different lags for cross-mapping are considered and the one producing the best mapping is selected. The given causal direction is accepted if the chosen lag is negative and rejected if it is positive. Extended CCM needs hundreds of consecutive gap-free regular-interval observations to use cross-validation in contiguous time intervals and to properly test convergence of the cross-map skill for different lags. For the data used, which has a time frequency of 8 days, this means 2-3 years of information are necessary for the standard and extended CCM. Since its introduction, the extended CCM has been applied in many different areas such as infectious diseases^53^, soil moisture and precipitation feedback analysis^8^ and the study of fish communities^54^ to name just a few.

#### Robust CCM (RCCM)

The direct application of the CCM method consists of a sequence of steps including hyperparameter selection for which user intervention is strictly required. Many choices need to be made, most involving heuristic criteria. This, in turn, yields quite unstable results depending on the run, initialization, selection of samples, etc. This hampers its wide adoption and applicability in practice. The proposed RCCM applies the extended CCM^21^ by using bootstrap sampling in order to improve robustness of the results while allowing automatic parameter selection. We implemented an automated pipeline based on the public available rEDM^55^ package supplied by the authors of the extended CCM (available at https://rdrr.io/cran/rEDM/man/CCM.html), for estimating hyperparameters. The crucial step consists of determining the dimension *p* of the embedding . Depending on its value, the ability for predicting the dynamics of one variable from the other’s varies significantly. To address this we exploited bootstrap resampling through time, and aggregated using the median in order to obtain more robust estimates of the cross-mapping skill $\tilde{\rho }$ in a spatially explicit way. Previous works have considered similar ideas yet in the spatial domain^56^.

The RCCM approach is applied in a pixel-wise manner, and works as follows. Regression parameters are selected by cross-validation-through-time: The first third of the series was used for training and the remaining for testing. Results were aggregated over $N=10$ runs where each run is generated by using time series with different starting and ending points. The time series were restricted to full years, and we controlled for the minimum number of consecutive non-empty observations needed. These time series are used as a surrogate bootstrapped ensemble to test the significance of the results. With such partitions, we computed the forecast skill for different length libraries using the extended CCM approach. Instead of looking for convergence in cross-map skill by looking at one estimate of the cross-map skill curve, we looked at an ensemble of cross-map skill difference curves (one time step difference) and aggregated across runs using the median to obtain a robust estimate of the change in cross map skill as library size increases , that is for $\rho _i$, $i=1,\ldots , N$. We also tried executing these two steps in reverse, but the results are less spatially smooth. Since we use 10 different train and test windows, more data is necessary with this robust approach. We used overlapping train and test windows in order to mitigate the amount of data necessary but even so we need around 400 regular-interval time observations spanning 9 years of information. Finally, for the chosen time dimension (library size), we evaluate the cross map skill using a set of different alignments between each pair of variables, so as to avoid false causal identification due to strong couplings. The optimal cross-map lag, denoted $t_p$, was selected with this criteria from a sequence ranging from -15 to 15 time steps (pre- to post- 4 months) with a step size of 1 for a total of 31 lags considered. The causal relationship was rejected if the $t_p > 0$. For non-positive lags, we estimated causal direction in combination with the following information-geometric method.

### Information-geometric causal inference (IGCI)

Information-Geometric Causal Inference (IGCI)^25^ tries to distinguish between cause and effect when two variables are involved only. Given two variables *X* and *Y*, the approach is based on an independence assumption between the distribution of the (potential) cause under scrutiny $P_X$ and the causal mechanism translating *X* into *Y*, that is the conditional *P*(*Y*|*X*). The original formulation of IGCI was proposed for the deterministic case where *X* and *Y* are related by an invertible (potentially nonlinear) function $Y = f (X)$ with $X = f^{-1}(Y)$.

The IGCI criterion is simple in practice. One has to measure the complexity of both possible causal directions and compute the difference as a causal criterion (sometimes referred to as the complexity loss), $C_{X}:=D(P_X|E_X) - D(P_Y|E_Y)$, where *D* measures the complexity of the distribution, and $E_X$ and $E_Y$ are the approximation residuals of *X* and *Y* respectively.

When the distance of functions *D* is chosen to be the relative entropy distance of the densities, several convenient simplifications emerge^25,57^. We used the Entropy-based IGCI, which infers $X\rightarrow Y$ whenever $H(P_X)>H(P_Y)$, where $\widehat{H}$ is an entropy estimator. The authors in^25^ suggested a particular entropy estimator in Ref. 58, but virtually any estimator can be used. We used the k-D partitioning tree-hierarchy (kDP) entropy estimation method^59^ . The causal direction $C_{X\rightarrow Y}$ is then given by $\widehat{C}_{X\rightarrow Y}:= \widehat{H}(P_Y)-\widehat{H}(P_X)$.

### Combining RCCM and IGCI

Both (R)CCM and IGCI are methods based on detecting asymmetries, work in bivariate (yet possibly multivariate) cause-effect pairs, and need to fulfil the conditions of faithfulness and sufficiency. They differ in that (R)CCM does not require the assumption of acyclic graphs. The combination of CCM and IGCI is not incidental, as both work for pairs of random variables. CCM and IGCI perform poorly in the presence of noisy observations, but there is some empirical evidence of performance in (moderate) noise regimes^21,24,25,60,61^. CCM performs poorly for strongly coupled variables, which can be compensated by IGCI when no strong confounder is present^57^. In the experiments in the paper, we use the IGCI approach to mask the RCCM results for cases that satisfied two coditions: 1) a non-negligible entropy difference, (i.e. $|\widetilde{C}_{X\rightarrow Y}|>\varepsilon =0.2$) and 2) instantaneous ($t_p=0$) or delayed causality ($t_p<0$) as estimated from the RCCM. Both RCCM aswell as IGCI and its combination are applied for each pixel individually.

### Robustness to noise and strong coupling

**Figure 8:**
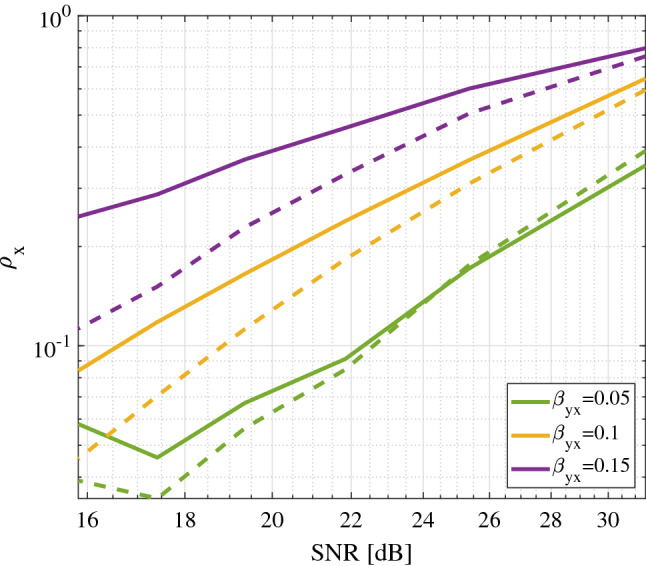
Cross-map skill $\rho _x$ as a function of noise in the effect variable $\sigma _y$ and level of coupling $\beta _{yx}$ in the coupled logistic map problem. The proposed combination of CCM and IGCI (solid lines) outperform the standard CCM (dashed lines), especially noticeable in high levels of noise and coupling.

Figure 8 shows the good robustness capabilities of the proposed combination of CCM and IGCI in a toy example involving the coupled logistic map, which is defined by $x_{t+1}=x_t(r_x(1-x_t)-\beta _{xy}y_t)$ and $y_{t+1}=y_t(r_y(1-y_t)-\beta _{yx}x_t)$. We followed^24^ and compared CCM (dashed lines) and the proposed CCM*IGCI (solid lines) for different coupling strengths $\beta _{yx}=\{0.05,0.10,0.15\}$, and noise variances $\sigma _y^2$ for the effect variable *Y*. We fixed both the time delay $t_p = 1$ and the embedding dimension $m=2$. Curves are the result of averaging 1000 runs using $N=1000$ samples. In all cases we considered the case of unidirectional coupling from *X* to *Y*, i.e., $\beta _{xy} = 0$ and noise-free cause $\sigma _x=0$. We fixed $r_x = 3.8$ and $r_y = 3.5$, so that *X* is in the chaotic regime and the dynamics of *Y* are governed by a period-4 attractor for the case that $\beta _{yx} = 0$. This makes the dynamics of *Y* increasingly chaotic as $\beta _{yx}$ increases. When the noise level $\sigma _y$ increases, the cross-mapped estimates of *X* from ${\mathcal M}_y$ deteriorate, and as a result $\rho _{x}$ decreases. Results show that the proposed approach is consistently improving the detection skill for all noise $\sigma _y$ and coupling $\beta _{yx}$ levels, especially noticeable as the noise or coupling increase.

**Figure 9:**
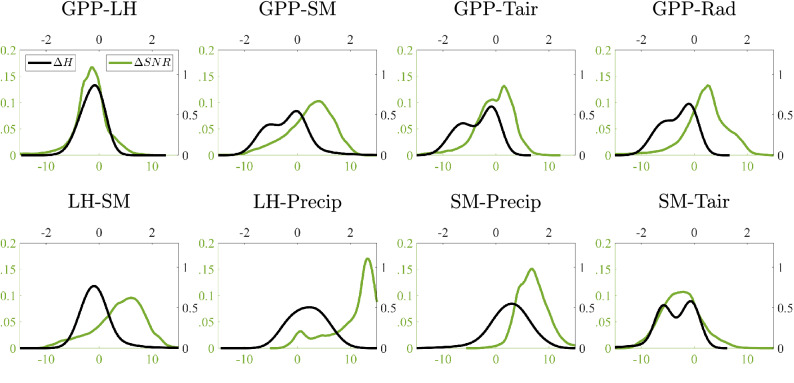
Densities of the differences in entropy, $\Delta$H (black lines), and signal-to-noise ratio, $\Delta$SNR[dB] (green lines) between the considered variables.

We illustrate the previous effects of coupling and noise on the considered variables too, see Fig. 9. Differences between the entropy of GPP versus meteorological variables ($\Delta$H$<0$, black lines) reflects the latter’s causal primacy. The biggest SNR differences ($\Delta$SNR$>0$, blue lines) are found for the SM and Precip variables, which explains the puzzling results when SM, and especially precipitation, are involved in the analysis, and justify the need for masking spurious relations. We noted that SNR differences do not seem to drive $\Delta$H but, in extreme cases, such as for precipitation, the use of the IGCI criterion could also break and provide unreliable results. In general, however, the combination of the two causal criteria provide good robustness capabilities in most of the cases.

## Data availability

All data are available via earthsystemdatalab.net or from the original data providers as indicated in the manuscript.
